# Improving pairwise sequence alignment accuracy using near-optimal protein sequence alignments

**DOI:** 10.1186/1471-2105-11-146

**Published:** 2010-03-22

**Authors:** Michael L Sierk, Michael E Smoot, Ellen J Bass, William R Pearson

**Affiliations:** 1 Bioinformatics Program and Chemistry Department, Saint Vincent College, Latrobe, PA 15650 USA; 2 Department of Medicine, University of California, San Diego, La Jolla, CA 92093 USA; 3 Department of Systems and Information Engineering, University of Virginia, Charlottesville, VA 22908 USA; 4 Department of Biochemistry and Molecular Genetics, University of Virginia, Charlottesville, VA 22908 USA

## Abstract

**Background:**

While the pairwise alignments produced by sequence similarity searches are a powerful tool for identifying homologous proteins - proteins that share a common ancestor and a similar structure; pairwise sequence alignments often fail to represent accurately the structural alignments inferred from three-dimensional coordinates. Since sequence alignment algorithms produce optimal alignments, the best structural alignments must reflect suboptimal sequence alignment scores. Thus, we have examined a range of suboptimal sequence alignments and a range of scoring parameters to understand better which sequence alignments are likely to be more structurally accurate.

**Results:**

We compared near-optimal protein sequence alignments produced by the Zuker algorithm and a set of probabilistic alignments produced by the probA program with structural alignments produced by four different structure alignment algorithms. There is significant overlap between the solution spaces of structural alignments and both the near-optimal sequence alignments produced by commonly used scoring parameters for sequences that share significant sequence similarity (E-values < 10^-5^) and the ensemble of probA alignments. We constructed a logistic regression model incorporating three input variables derived from sets of near-optimal alignments: robustness, edge frequency, and maximum bits-per-position. A ROC analysis shows that this model more accurately classifies amino acid pairs (edges in the alignment path graph) according to the likelihood of appearance in structural alignments than the robustness score alone. We investigated various trimming protocols for removing incorrect edges from the optimal sequence alignment; the most effective protocol is to remove matches from the semi-global optimal alignment that are outside the boundaries of the local alignment, although trimming according to the model-generated probabilities achieves a similar level of improvement. The model can also be used to generate novel alignments by using the probabilities in lieu of a scoring matrix. These alignments are typically better than the optimal sequence alignment, and include novel correct structural edges. We find that the probA alignments sample a larger variety of alignments than the Zuker set, which more frequently results in alignments that are closer to the structural alignments, but that using the probA alignments as input to the regression model does not increase performance.

**Conclusions:**

The pool of suboptimal pairwise protein sequence alignments substantially overlaps structure-based alignments for pairs with statistically significant similarity, and a regression model based on information contained in this alignment pool improves the accuracy of pairwise alignments with respect to structure-based alignments.

## Background

Pairwise sequence alignment is the most widely used method for extracting information from protein and DNA sequences; it is routinely used to detect protein homologs that diverged more than 2 billion years ago. Homology -common evolutionary ancestry - can be reliably inferred for proteins that share statistically significant sequence similarity. When statistically significant similarity to a known sequence is found, inferences can be made about the structure, function, and biologically significant residues of the unknown sequence. While the inference of homology is quite robust (proteins that share significant similarity in pairwise alignments always have similar structures), [1] some of the more detailed functional inferences are critically dependent upon the quality of the alignment between the two sequences. For proteins that are very similar (>60% identity), functional inferences are usually very accurate, but for more distantly related proteins, ambiguity in the alignment of poorly conserved regions can lead to errors [2].

**Figure 1:**
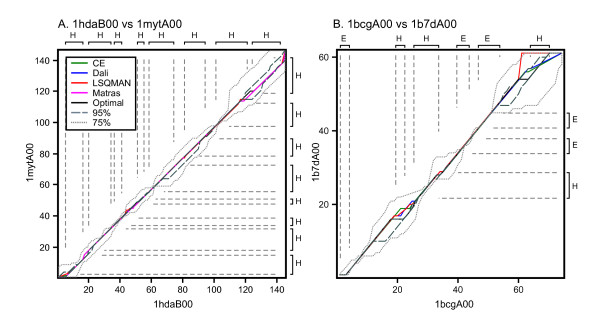
**Alignment paths of structure-based, and optimal and suboptimal sequence alignments**. Two pairs of aligned proteins are shown: (A) 1 hdaB00 vs 1 mytA00, pair 25 in Figure 4, 26.8% identity, E() 2.1 × 10^-10^; and (B) 1bcgA00 vs. 1b7dA00, pair 42 in Figure 4, 33.3% identity, E() = 9.6 × 10^-7^. Four structural alignments are shown: CE (green), DALI (blue), LSQMAN (red), and Matras (magenta). Also shown is the best global sequence alignment (black, scoring matrix BLOSUM50, gap penalties -10/-2), the envelope of alignments within 95% of optimal (grey, dashes), and the envelope of alignments within 75% of optimal (light grey, dots). The Zuker suboptimal alignment algorithm produced 2 optimal alignments for 1 hdaB00:1 mytA00, 45 alignments at 95% optimal, and 1,170 at 75% optimal. For 1bcgA00:1b7dA00 there were 8 optimal, 34 95% optimal, and 349 75% optimal alignments produced. Both protein pairs are from the medium sequence similarity group. The axes and vertical/horizontal dashed gray lines highlight the secondary structure elements as defined by the PDB file.

The usual gold standard by which sequence alignments are assessed is the structural alignment between two proteins whose three-dimensional (3D) structures are known. The 3D-structure contains more information than the one-dimensional sequence, and diverges more slowly, so that distant evolutionary relationships can be recognized in structures between sequences that do not share statistically significant similarity. However, even clearly related proteins with strong sequence similarity can produce sequence alignments that differ from the most accurate structural alignments (Figure 1). Since determining the three-dimensional structure of every protein is not feasible, we seek strategies to produce structurally accurate homology models for sequences of unknown structure. The most common and successful methods involve finding a template among the set of known structures upon which to base the model. In the case of high sequence similarity (i.e. >60% identity), this task is relatively trivial, since the sequence and structural alignments are generally quite similar in this range. However, there are only a limited number of sequences that can be modeled in this region; there are far more sequences in the so-called "twilight zone" (i.e. ~20-40% sequence identity) where divergent, but clearly homologous proteins can be difficult to align. Since the quality of the final 3D-model depends on the alignment of the unknown sequence to the structural template, we focus on improving the quality of alignments between proteins that share statistically significant similarity, and 20% to 40% sequence identity [3,4].

The most widely used algorithm for generating pairwise sequence alignments is dynamic programming, originally applied to biological sequences by Needleman and Wunsch[5]. Dynamic programming methods guarantee an algorithmically optimal alignment for the specific sequence and input parameters. However, an optimal sequence alignment score does not necessarily guarantee that the alignment is structurally accurate. Sequence alignment scores are optimized for a particular set of amino acid replacement scores and gap penalties; there is no natural process by which two proteins align themselves. For this reason "optimal" sequence alignments can be very different from optimal structural alignments. For example, in Figure 1A, the highest scoring structural alignment (produced by LSQMAN) had a semi-global sequence alignment score of -20, which is dramatically lower than the optimal sequence alignment score of 145. Yet the actual LSQMAN alignment is very similar to the other structural alignments, and the optimal sequence alignment. The LSQMAN sequence alignment score is very low because LSQMAN has a number of gaps of the form ACD--/---TQY, which would never be produced in a sequence alignment, but may make sense (because two loops are geometrically distant) in a structural alignment.

Furthermore, distantly related proteins often have multiple optimal alignments, as well as a large number of suboptimal alignments with scores very close to that of the optimal score [4,6-8]. As one moves away from the optimal score, the number of alternative alignments grows rapidly. Thus, to keep the number of alignments to be dealt with computationally tractable, one must sample the suboptimal alignment space.

Although the "gold standard" by which sequence alignments are evaluated is a structure-based alignment, structural alignments can vary, and there is no algorithm that guarantees an optimal structural alignment [9]. The assumption is usually made that because structures tend to vary less than sequences over evolutionary time, differences in structural alignments are small compared to the difference between sequence and structural alignments. While this is certainly true for very distantly related proteins that do not share significant similarity (and thus cannot be meaningfully aligned from sequence data alone), the range of structural and sequence alignment accuracy has not been carefully examined for proteins that share statistically significant similarity. Our results suggest that for proteins with moderately significant sequence similarity, sequence alignments can often be within the range of different structural alignments.

Since structurally accurate alignments often have sub-optimal sequence alignment scores, investigators have explored these sets of alternative alignments, asking whether they provide information about accurate structural alignments. For example, Jaroszewski et al. [4] examined alternative alignments generated both from a near-optimal alignment generation algorithm and by varying the scoring parameters (i.e. the gap penalties and substitution matrix) and showed that there is frequently an alignment in these sets that is closer to the structural alignment. They concluded that the two methods of generating alternative alignments - alternative scoring parameters and sub-optimal alignment - have complementary (as opposed to redundant) information, since the union of the two sets yielded many more alignments that matched a structural alignment than either of the single sets. Holmes and Durbin [10] also investigated the accuracy of the optimal sequence alignment and developed a method for calculating the expected accuracy of a given alignment. Zhang and Marr [11] used an algebraic approach to investigate alternative alignments in the neighborhood of the optimal alignment.

Various authors have taken a probabilistic approach to generating sets of alternative alignments. Miyazawa [17] calculated alignment probabilities based on the exponent of the alignment score, and compared the resultant probabilities of matched amino acids in the alignment to the respective protein structure alignments. Yu and Hwa examined the statistical significance of alignments produced using a pairwise Hidden Markov Model (HMM) [12]. Knudsen and Miyamoto [13] developed an alignment method based on a pairwise HMM that included an explicit evolutionary model for indels. Finally, Mückstein et al. [14] developed a procedure for sampling alignments based on statistical weighting using the partition function over all possible alignments of two sequences.

While it is of theoretical interest to compare sets of individual sequence and structure alignments, it is only of practical use if one can determine which sequence alignment is the correct one in the absence of structural information. One way to address this question is to estimate the reliability of a particular pair of aligned residues (which we call an edge, using the convention that in the dynamic programming path graph, aligned residues, insertions, and deletions are scored along edges, while the optimal score is calculated at the vertex). Cline et al. [15] looked at four methods for predicting the reliability of a particular pair of aligned residues and determined that the method proposed by Yu and Smith [16] for extracting near-optimal alignments from a profile Hidden Markov Model (HMM) provided the most improvement in alignment quality. Miyazawa [17], Knudsen and Miyamoto [13], and Mückstein et al. [14] examined the relationship between edge probabilities and structural alignment, although in the latter two cases only in the context of a small number of protein pairs, and generally found a good correspondence between them. Mevissen and Vingron [18] demonstrated the efficacy of an edge reliability index called robustness, which had been defined previously by Chao et al. [19], among others (see Methods). They demonstrated that the robustness of an edge accurately predicted whether the edge was also aligned in the structural alignment. Here, we extend the analysis of robustness by incorporating additional alignment quality information and developing a logistic regression model that returns (via the logit link function - see Methods) the probability that a given edge is contained in a structural alignment. We also examine the distribution of alignments produced by probA (Mückstein et al. [14]) and compare them to the distribution produced by the Zuker algorithm [8].

## Results

Our goal is to find characteristics of sub-optimal sequence alignments that can be used to identify alignments, or sub-alignments, that are found in structural alignments. Just as importantly, we seek measures of alignment quality that help us predict which alignments are more likely to be correct. Since homologous proteins are typically identified through similarity searches, we focus on protein sequences that share statistically significant sequence similarity. We divide our protein pairs by sequence similarity, to explore the relationship between sequence similarity (statistical significance) and structural accuracy. The most similar third of the alignment pairs have sequence similarity expectation values E() < 10^-10^, with an average of 48% identity. The intermediate and most distantly related sequences have 10^-10 ^< E() < 10^-5 ^(26.9% identity) and E() < 10^-5 ^(22.6% identity), respectively.

### Comparison of Near-Optimal and Structure-based Sequence Alignments

Suboptimal sequence alignments can only be used to produce accurate structural alignments if the sequence and structure alignments overlap each other. Figure 1 depicts the sequence/structural alignment overlap in the context of the path graph. In Figure 1A, the alignment of two globin homologs, the structure-based alignments tend to overlap extensively, with only minor deviations from each other, in large part because the eight globin α-helices comprise most of the sequence. The optimal sequence alignment also overlaps the structure alignments extensively. The lines for the 95% of optimal and 75% of optimal sets represent envelopes: i.e. for a given residue in the path graph, the two lines mark the maximum and minimum indices of the residues it is aligned against in the pool of alternative alignments. In Figure 1B, two neurotoxins that have a set of three conserved secondary structural elements, but other substantial structural differences, the structural alignments are less consistent, and the 75%-optimal sequence alignments are required to capture the structural uncertainty. In both cases, the structural alignments are contained within the set of alternative sequence alignments.

**Figure 2:**
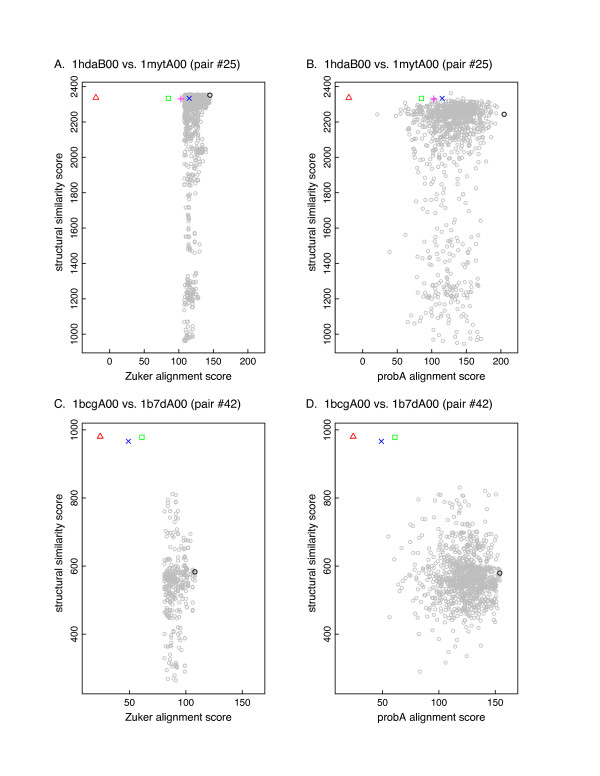
**Structural and sequence similarity scores of near-optimal sequence alignments and structural alignments for the protein pairs shown in Figure 1**. Figures A. and B., 1 hdaB00 vs 1 mytA00 (pair 25). Figures C. and D., 1bcgA00 vs. 1b7dA00 (pair 42). Figures A. and C. show Zuker-generated alignments within 95% of the optimal score, using the BLOSUM50 scoring matrix and gap open/extension penalties of -10/-2. Figures B. and D. show 1000 probA-generated alignments using the BLOSUM50 scoring matrix, and gap open/extension penalties of -9.5/-1.2. The X-axis shows the semi-global sequence alignment score; the Y-axis shows the corresponding Levitt-Gerstein structural similarity score. Optimal sequence alignment, black circle; suboptimal alignments, gray circles. Blue X, Dali; green square, CE; red triangle, LSQMAN; magenta +, Matras. The optimal sequence alignment in Figure 2A has a structural similarity score of 2352, higher than any of the structure-based alignment scores (RMSD 1.51 Å, shift score of 0.94 with respect to the DALI alignment). The structural alignments have structural similarity scores ranging from 2330 to 2337, with RMSDs of 1.47 Å. In Figure 2C, the optimal sequence alignment has a structural alignment score of 2167 (RMSD 1.33 Å, shift score with respect to DALI of 0.66). Structural alignments have structural alignment scores ranging from 2210 to 2274, with RMSDs from 1.35 - 1.38Å. Matras did not produce an alignment for 1bcgA00 vs. 1b7dA00.

Alignments have a score - either a sequence similarity score or a structural similarity score. While the path graphs indicate that the structural alignments are generally in the space of suboptimal sequence alignments, we need to know how the similarity scores compare. Figure 2 shows the sequence and structural similarity scores for the alignments depicted in Figure 1 (see Methods for details). In Figure 2A, the best sequence alignments have structural similarity scores that are as good as or better than those of the structural alignments. In Figure 2C the best sequence alignments have structural similarity scores that are about 20% lower than the structure-based alignments. Moreover, while the structure-based alignments tend to have similar structural similarity scores, their sequence similarity scores vary considerably. The alignments produced by probA are shown in Figures 2B and 2D. The probA sequence alignment scores span a wider range than the Zuker alignments, but this does not necessarily result in alignments with higher structural similarity scores. Table 1 summarizes the alignment scores for the two pairs shown in Figures 1 and 2.

**Table 1:** Sequence and structural alignment scores for two example alignments.

	Levitt-Gerstein Structural Similarity	Sequence Alignment Score^†^	Shift Score vs. Dali	Levitt-Gerstein Structural Similarity	Sequence Alignment Score^†^	Shift Score vs. Dali
**Alignment**	1 hdaB00 vs. 1 mytA00 (pair 25)			1bcgA00 vs. 1b7dA00 (pair 42)		

CE	2333	85	0.986	978	61	0.920
Matras	2330	103	0.983	966	49	-
DALI	2334	115	1.000	980	24	1.000
LSQMAN	2337	-20	0.946	980	24	0.940
Optimal Sequence^§^	2352	145	0.940	583	108	0.837
95% Neighborhood^§^	2355	145	0.900	696	104	0.880
75% Neighborhood^§^	2359	145	0.923	812	88	0.780
probA^§^	2362	37	0.645	830	37	0.757
robustness^¶^	2301	-526	0.734	383	24	0.565
model^¶^	2352	145	0.941	579	108	0.796

^†^Semi-global alignment, Blosum50 substitution matrix, gap open penalty -10, gap extension penalty -2.

^§ ^The sequence with the highest Levitt-Gerstein similarity score was chosen from the set of optimal alignments, the set of alignments with a sequence similarity score within 95% of optimal, the set of alignments with a sequence similarity score within 75% of optimal, and the set of probA alignments, and the sequence similarity score and shift score for that alignment were recorded.

^¶^Alignments were created using the log-odds score produced by a logistic regression model in place of a substitution matrix. "Robustness" refers to a model that had only robustness as an independent variable, while "model" refers to the full model (incorporating robustness, edge frequency, and maximum bits-per-position). See text for details.

**Figure 3:**
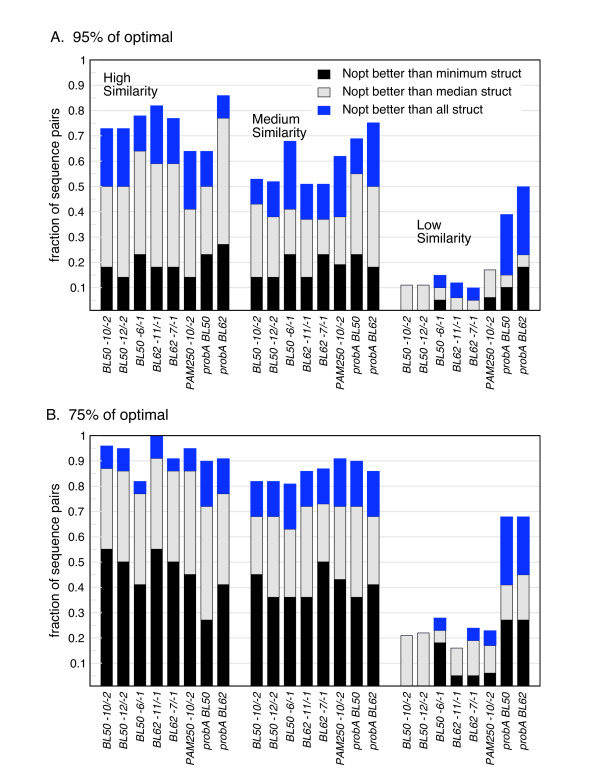
**Comparison of near-optimal sequence alignments to structural alignments**. (A) Sampled sequence alignments within 95% of the optimal score (for Zuker alignments), or the top 100 alignments (for probA); (B) sampled sequence alignments within 75% of the optimal score (for Zuker alignments), or the top 500 alignments (for probA). The X-axis has been divided into three groupings, representing three levels of sequence similarity: high (E() < 10^-10^), medium (E() < 10^-5^), and low (E() < 0.02). Within the similarity groups, different combinations of scoring matrices and gap penalties are shown. The Y-axis reports the fraction of protein families with a near-optimal sequence alignment that has a structural similarity score better than the best structural alignment (blue), the median structural alignment (grey), and the lowest structural alignment score (black). Structural alignments were not used if the number of aligned residues was less than 50% of the maximum number of aligned residues for that pair.

Figure 3 summarizes the analysis shown in Figure 2 for 66 pairs of sequences whose structures are known, across a range of scoring matrices and gap penalties. In addition to scoring matrices and gap penalties that are widely used for sequence similarity searching (BL50, -10/-2, BL62, -11/-1), we examined some much lower gap penalties, since we observed the "unusual" gapping patterns in the structural alignments discussed above. The data are broken down into three similarity levels, according to expectation values. Figure 3A shows the results of the 95% of optimal set (for the Zuker alignments) or the top 10% of alignments (for the probA alignments), and Figure 3B the results for the 75% of optimal set (for the Zuker alignments) or the top 50% of alignments (for the probA alignments). Sequence alignments with scores within 95% of optimal have structural similarity scores as high as the structural alignment with the lowest score in more than 60% of pairs for the most similar sequences we examined (E() < 10^-10^) and in more than 50% of pairs for moderately similar sequences (E() < 10^-5^). Indeed, 95% sub-optimal sequence alignments were more accurate than the best structural alignment in 10%-20% of the families in both similarity groups. When a sampling of sequence alignment scores that are within 75% of optimal are examined (Figure 3B), a sequence alignment as structurally accurate as the least accurate structure alignment is produced in more than 80% of both the most similar pairs and the moderately similar pairs. However, for sequences that share less similarity (E() < 0.02 and E() > 0.02), sub-optimal sequence alignments rarely include structurally high-scoring alignments. Also shown in Figure 3 are the results for the probA alignments, using either BLOSUM 50 or BLOSUM 62. In contrast to the examples in Figure 2, the wider sampling produced by probA does result in an increase in the number of alignments with good structural similarity scores, particularly in the low similarity group.

Figure 3 demonstrates that there is substantial overlap between the set of near-optimal alignments and the four structural alignments, especially for the pairs of sequences that are most highly similar to each other (as is to be expected). They also demonstrate that increasing the size of the pool of near-optimal alignments increases the probability of finding a near-optimal alignment as good as or better than a structural alignment. In addition, Figure 3 indicates that there is no set of scoring parameters that is clearly better than the others in terms of producing high-scoring structural alignments, for the six scoring matrix/gap penalty combinations examined. Finally, it is instructive to note the difference between the medium and low sequence similarity groups: the former has E()-values between 10^-10 ^and 10^-5 ^and a mean percent identity of 26.9% (median 25.2%), while the latter has E()-values between 10^-5 ^and 0.02 and a mean percent identity of 22.7% (median 22.1%). There is clearly a significant drop-off in alignment quality going from the medium similarity group to the low similarity one, even though the average percent identity does not change very much. The expectation value is much more informative predictor of sequence alignment accuracy than the percent identity, even for sequences that share significant similarity.

**Figure 4:**
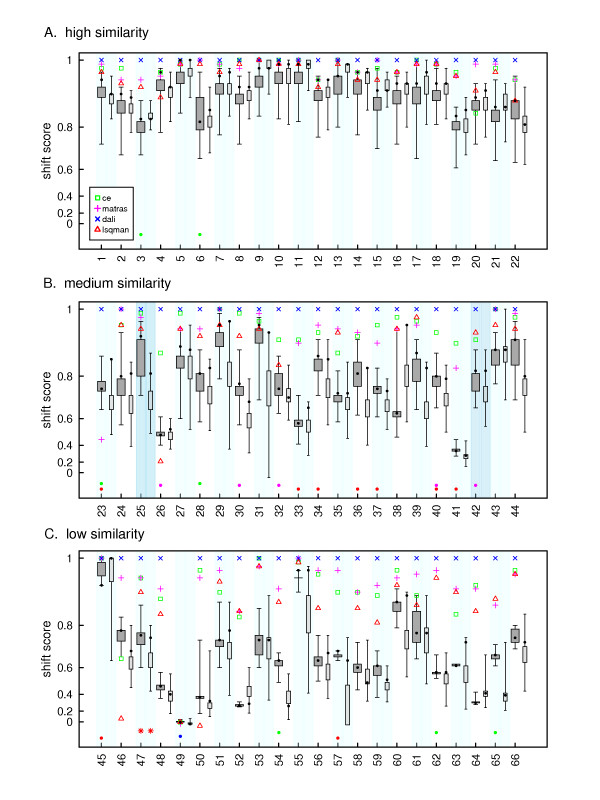
**Boxplot depicting the distribution of shift scores for structural alignments and near-optimal sequence alignments**. Shift scores are calculated relative to the Dali alignment for each pair of proteins. Shift scores, which range from -0.2 to 1.0, were plotted on a logarithmic scale to highlight the region between 0.6 and 1.0. DALI shift scores (the reference) are plotted as blue X's, CE as green squares, Matras as magenta +'s, and LSQMAN as red triangles. The Zuker near-optimal alignments are those with a similarity score within 75% of the optimal score. The boxplot for 1000 probA alignments is plotted to the right of the Zuker boxplots for each pair. The three panels present the groups of proteins that share high, medium, and low statistically significant sequence similarity. Protein pairs within a similarity group are ordered from left to right by expectation value (i.e. most similar to least similar). Pairs 25 and 42 from Figures 1 & 2 are highlighted with a darker blue rectangle. The colored dots at the bottom of each column represent either very poor or nonexistent alignments produced by the structural alignment programs. The colors match those of the symbols for the high quality alignments.

A single similarity score is only a rough measure of how similar two alignments are. Cline et al. [15] developed the shift score as more robust measure of how similar two alignments are, and demonstrated its ability to account for both shorter alignments that are highly similar and longer alignments that have several edges that are different by a few residues. Figure 4 shows the distribution of shift scores for near-optimal alignments (75% neighborhood for the Zuker alignments, 1000 alignments for the probA alignments) with respect to a single structure alignment (that produced by the Dali program). As in Figures 2 and 3, the substantial overlap between near-optimal alignments and structural alignments is apparent for the most similar sequences, although the two begin to diverge as the sequence similarity drops. The structural alignments also become more divergent from each other as the sequence similarity drops, and, perhaps surprisingly, 6 of the 22 aligned pairs of medium similarity have at least one (Zuker) sequence shift score as high or higher than a structure-based alignment, and 18 have alignments within 0.2 of the lowest structural alignment (excluding structure alignments that aligned fewer than half of the number residues aligned by the Dali alignment, and cases where the program did not produce an alignment). For the low similarity pairs, 2 of the 22 pairs have sequence alignments as good as or better than the lowest structural alignment, and 9 are within 0.2 shift scores of a structural alignment. Thus, there is still substantial overlap between the near-optimal sequence alignment space and the structural alignment space, even using the more sensitive shift score. Consistent with Figure 3, the probA alignments produce wider ranges of shift scores, which sometimes results in overlap with the lowest structural alignment. However, there does not appear to be any consistent pattern when comparing the Zuker and probA alignments. There are examples where most of the Zuker distribution is higher than most of the probA distribution (e.g. pair 25); where the probA distribution is higher (e.g. pair 38); where the optimal Zuker alignment (highlighted by a black circle) has a better shift score than the best probA alignment (e.g. pair 39); and where the best probA is better (e.g. pair 23). It does appear that the best probA alignment has the best shift score of all the probA alignments (e.g. pair 38) more frequently than the optimal Zuker alignment has the highest shift score, but this occurs in only 8 out of 66 pairs.

### Logistic Regression Model

To make use of the information contained within the set of near-optimal alignments, one needs a way of ranking or assessing the alignments. In particular, one would like to assess the likelihood that a particular pair of residues has been aligned properly. Mevissen and Vingron described one such method [18]. They calculated the robustness, which is the difference between the sequence alignment score for an alignment including a given pair of aligned residues (i.e. an edge in the path graph of the alignment) and the highest score for an alignment that does not include that edge. They demonstrated that robustness reasonably predicted the reliability that the edge would be found in the structural alignment. Robustness gives a measure of the importance of a given edge, but it does not measure aspects of the overall alignment. The frequency that an edge is found in a set of near-optimal alignments incorporates the fact that the area in the path graph surrounding the edge in question may affect whether that particular edge is used frequently. In addition, the maximum bits-per-position score that is obtained by an alignment containing that edge in the set of near-optimal alignments provides information about the overall quality of the alignments that use that edge.

Logistic regression is used to predict a discrete response variable using one or more continuous, discrete, and/or dichotomous predictor variables [20]. Thus we developed a logistic regression model using robustness, frequency, and maximum bits-per-position as predictors, and presence in a structural alignment as the response. As detailed in the Methods section, sequence alignment scoring parameters, the threshold for structural targets, and the edge sample size did not substantially affect the resultant model. This is consistent with Figure 3, which indicates that altering the scoring parameters did not substantially affect the overlap between the set of alternative alignments and the structural alignments. We also examined different cutoffs for the neighborhood of the optimal score from which alignments were chosen (75%, 85%, and 95% of optimal). The ROC curves and AIC values improved the further the cutoff was from optimal. We also compared using all edges vs. edges from within the local alignment boundaries (see below), and found that the latter gave better AIC values. The final model presented in Tables 2 and 3 was constructed from 5000 randomly selected edges taken from the local region of alignments within 75% of optimal, and generated using three different scoring parameters: BLOSUM50 (-10 gap open penalty/-2 gap extension penalty), BLOSUM50 (-12/-2) and BLOSUM 62 (-11/-1).

**Table 2:** Logistic Regression Model

	Estimate	Std. Error	Wald Z-score	p-value
(Intercept)	-6.1032	0.3614	-16.888	< 2e-16
Frequency	5.7816	0.3904	14.808	<2e-16
Robustness	4.7489	0.8787	5.405	6.49e-08
Maximum bits- per-position	-1.6225	0.5842	-2.777	0.00548

The final logistic regression model parameters, based on edges drawn from the set of alignments with sequence similarity scores within 75% of optimal, limited to the local alignment region. See text for details of the model construction.

**Table 3:** Analysis of variance for logistic regression model

	Degrees Freedom	Deviance	Residual Degree Freedom	Residual Deviance	p-value
NULL			4999	1895.85	
Frequency	1	1369	4998	526.88	1.158e-299
Robustness	1	23.6	4997	503.24	1.164e-06
Maximum bits-per-position	1	7.7	4996	495.51	0.01

AIC	503.5				

See text for details of the model construction. AIC, Akaike Information Criterion.

**Figure 5:**
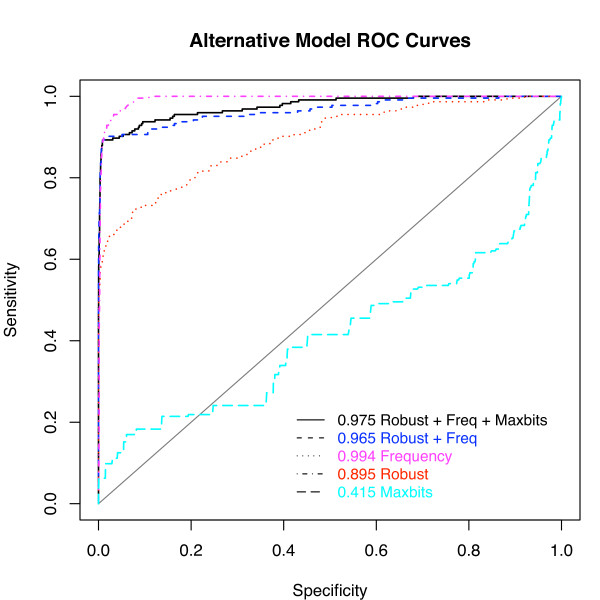
**Comparison of various logistic regression models in identifying structurally accurate alignment edges using the Receiver Operator Characteristic (ROC)**. The test set of 5000 edges (see Methods) from the 75% of optimal neighborhood was ranked by the log-odds score produced by the full logistic regression model, by a model using just frequency and robustness, and by models using the three variables independently. If a particular edge was found in 2 out of the 4 structural alignments, it was considered a true positive. (Using different thresholds for true positives did not substantially affect the performance.) The x-axis plots the probability of a false positive, while the y-axis plots the probability of a true positive. Curves higher and further to the left do a better job predicting whether an edge will be found in a structural alignment. The area under the curve (AUC) is reported for each of the models used.

We compared the ability of the model to predict whether an edge will be found in a structural alignment or not with that of alternative models (incorporating just frequency and robustness, or just the individual predictor variables) by creating training and test sets of edges by randomly segregating the alignments, then selecting edges from the pool of training or testing alignments. Figure 5 shows the Receiver-Operator Characteristic (ROC) curves for the various models. The area under the curve (AUC) improves from 0.895 to 0.975 using the logistic regression model compared to using robustness alone. This particular ROC shows a higher AUC using only frequency than using the full model, but the AIC values in Table 4 indicate that the full model provides the best fit to the data (see Methods), and the application of the different models indicates that the full model performs slightly better than using frequency alone (see below).

**Table 4:** Residual Deviance and AIC values for different regression models.

Model	Residual Deviance	AIC
Full Model	495.5	503.5

Freq + Robust	503.2	509.2

Frequency	526.9	530.9

Robustness	1038.0	1042.0

Since the probA alignments sampled a wider variety of alignments, which tended to overlap with the structural alignments to a greater extent than the Zuker alignments did, we also created a model using the probA alignment edges as input. This model performed slightly worse than that based on the Zuker alignments did (AUC 0.942, AIC 1327). We did not directly examine the edge probabilities that probA uses to construct its alignments, but since the algorithm produces a "statistically weighted distribution" [14] of alignments, the frequency of an edge should approximately correspond with its probability. Thus if the probA edge probabilities were substantially better at correctly predicting structural edges this should show up in the regression. The implication is that probA expands the diversity of alignments compared to the Zuker algorithm, but it is not necessarily any easier to identify which edges are found in structure alignments.

### Applying the model

Having established that the model is superior at identifying structural edges, we next wanted to make use of this information to produce more structurally accurate alignments. We did this in two ways: by "trimming" the optimal sequence alignments and by generating new alignments using the model probabilities.

Given a sequence alignment, some fraction of the aligned residues will be incorrect, compared to a reference alignment. (Here we used the Dali alignments as a reference, although in principle any of the structure-based sequence alignments could have been used.) Furthermore, some fraction of the correct edges will not appear in the sequence alignment. Perhaps the simplest way to make use of an edge classification scheme is to identify suspect edges, and remove them from the alignment. We thus "trimmed" alignments by removing edges from the alignment with low log-odds probabilities produced by the model, or with low robustness or frequency scores. For example, we replaced any edges in an alignment with low log-odds scores (e.g. < 0.5) with a gapped alignment (e.g. A/V becomes A-/-V). We then compared the percentage of sequence alignment edges that are correct (true positives) and the shift scores against Dali alignments of "trimmed" alignments with the untrimmed alignments. These results are summarized in Tables 5 and 6.

**Table 5:** Shift Score Summary

	Alignments	Better (count)	Worse (count)	Better (avg. (stdev))	Worse (avg. (stdev))
High Similarity	model trim vs. optimal	3	1	0.011 (0.005)	-0.009 (0.000)
	
	robust trim vs. optimal	3	1	0.010 (0.006)	-0.009 (0.000)
	
	frequency trim vs. optimal	3	1	0.010 (0.006)	-0.009 (0.000)
	
	local trim vs. optimal	13	0	0.022 (0.017)	0
	
	local trim + model trim vs. local	0	10	0	-0.008 (0.003)
	
	model alignment vs. optimal	8	3	0.025 (0.035)	-0.008 (0.002)
	
	robust alignment vs. optimal	3	12	0.019 (0.014)	-0.140 (0.130)
	
	frequency alignment vs. optimal	6	3	0.028 (0.038)	-0.008 (0.002)

Medium Similarity	model trim vs. optimal	7	3	0.036 (0.025)	-0.013 (0.006)
	
	robust trim vs. optimal	9	4	0.032 (0.025)	-0.010 (0.005)
	
	frequency trim vs. optimal	7	4	0.037 (0.025)	-0.011 (0.006)
	
	local trim vs. optimal	14	2	0.088 (0.076)	-0.014 (0.001)
	
	local trim + model trim vs. local	6	10	0.019 (0.010)	-0.017 (0.013)
	
	model alignment vs. optimal	10	2	0.034 (0.030)	-0.016 (0.013)
	
	robust alignment vs. optimal	2	19	0.095 (0.062)	-0.200 (0.150)
	
	frequency alignment vs. optimal	9	2	0.035 (0.030)	-0.016 (0.013)

Low Similarity	model trim vs. optimal	7	3	0.032 (0.022)	-0.022 (0.015)
	
	robust trim vs. optimal	11	3	0.027 (0.020)	-0.022 (0.016)
	
	frequency trim vs. optimal	7	3	0.032 (0.022)	-0.022 (0.015)
	
	local trim vs. optimal	18	3	0.100 (0.096)	-0.240 (0.390)
	
	local trim + model trim vs. local	5	8	0.023 (0.019)	-0.013 (0.007)
	
	model alignment vs. optimal	7	2	0.025 (0.014)	-0.026 (0.019)
	
	robust alignment vs. optimal	3	16	0.088 (0.066)	-0.220 (0.180)
	
	frequency alignment vs. optimal	6	3	0.023 (0.014)	-0.024 (0.014)

Numbers shown are the number of sequence pairs, out of 22 in each similarity group, whose shift score with respect to the Dali alignment are improved or worsened by at least 0.005, along with the average and standard deviation of the magnitude of the change in shift score. In the case of local alignments or alignments limited to the local alignment boundaries, the Dali alignment is also limited to the local alignment boundaries. Model trim is the optimal alignment, with edges that fall below 50% probability according to the logistic regression model removed. Robust trim is the same, except edges less than a normalized robustness score of 0.5 are removed. Frequency trim is for edges with a frequency less than 0.5 removed. Local trim is the optimal (semi-global) alignment, with all edges outside the SSEARCH boundaries removed. Model alignment is an alignment produced using the log-odds score produced by the full logistic regression model in place of the substitution matrix scores. Robust and frequency alignment is the same, except the logistic regression model only used robustness or frequency as a variable.

**Table 6:** True Positive Summary

	Alignments	Better (count)	Worse (count)	Better (avg. (stdev))	Worse (avg. (stdev))
High Similarity	model trim vs. optimal	10	0	0.018 (0.014)	0
	
	robust trim vs. optimal	8	0	0.019 (0.014)	0
	
	frequency trim vs. optimal	11	0	0.015 (0.013)	0
	
	local trim vs. optimal	15	0	0.024 (0.016)	0
	
	local trim + model trim vs. local	4	6	0.013 (0.010)	-0.009 (0.003)
	
	model alignment vs. optimal	8	3	0.024 (0.031)	-0.008 (0.003)
	
	robust alignment vs. optimal	3	12	0.024 (0.013)	-0.170 (0.170)
	
	frequency alignment vs. optimal	8	1	0.022 (0.030)	-0.012

Medium Similarity	model trim vs. optimal	14	1	0.056 (0.058)	-0.009
	
	robust trim vs. optimal	14	0	0.061 (0.056)	0
	
	frequency trim vs. optimal	13	1	0.053 (0.057)	-0.009
	
	local trim vs. optimal	14	3	0.098 (0.084)	-0.012 (0.005)
	
	local trim + model trim vs. local	9	8	0.061 (0.056)	-0.015 (0.009)
	
	model alignment vs. optimal	10	2	0.039 (0.043)	-0.020 (0.018)
	
	robust alignment vs. optimal	2	19	0.062 (0.000)	-0.250 (0.140)
	
	frequency alignment vs. optimal	8	2	0.045 (0.043)	-0.020 (0.018)

Low Similarity	model trim vs. optimal	12	1	0.054 (0.066)	-0.024
	
	robust trim vs. optimal	17	0	0.051 (0.055)	0
	
	frequency trim vs. optimal	12	1	0.055 (0.066)	-0.024
	
	local trim vs. optimal	18	2	0.110 (0.100)	-0.280 (0.380)
	
	local trim + model trim vs. local	10	3	0.041 (0.065)	-0.013 (0.005)
	
	model alignment vs. optimal	7	2	0.021 (0.014)	-0.034 (0.022)
	
	robust alignment vs. optimal	2	16	0.150 (0.050)	-0.240 (0.170)
	
	frequency alignment vs. optimal	5	3	0.022 (0.016)	-0.029 (0.018)

Numbers shown are the number of sequence pairs, out of 22 in each similarity group, whose percentage of aligned edges that are correct with respect to the Dali alignment are improved or worsened by at least 0.005, along with the average and standard deviation of the magnitude of the difference. In the case of alignments limited to the local alignment boundaries, the Dali alignment is also limited to the local alignment boundaries. Alignments are as described in Table 5.

Trimming the alignments with the logistic regression model probabilities clearly improves the percentage of true positives in all similarity categories, in agreement with the ROC curve. This has minimal effects on the sensitivity (coverage); of the edges in the optimal semi-global alignment removed by the model, less than 10% (49/503) are true positives (i.e. are aligned in 2 out of 4 structural alignments). No more than four true positives are removed in any given pair. For shift scores, trimmed alignments also show clear, if less dramatic, improvement over the optimal alignment. In this trimming analysis, the full model performs similarly to robustness alone and frequency alone.

Another way to look at alignment quality is to identify regions of the alignment that are of higher or lower quality, as opposed to individual edges. Since we are aligning structurally defined domains, the default was to use semi-global alignments; however, we hypothesized that the regions of the alignment that were within the local alignment boundaries might be of higher quality, under the assumption that there would be more alignment "signal" within this region. Tables 5 and 6 indicate that this is indeed the case, since when we trimmed the optimal global alignments (using the same procedures as described above) to the local alignment boundaries (determined by SSEARCH alignments), the true positives and shift scores improved significantly. The semi-global alignments trimmed to local boundaries perform better than the SSEARCH alignments, indicating that the global alignment is necessary even though it is typically of poorer quality at the ends of the alignment (data not shown). Combining trimming by both local boundaries and the regression model did not result in better performance, indicating that a significant portion of the improvement due to the model trimming is due to removing incorrect edges that are at the ends of the alignment (see Tables 5 and 6).

Trimming alignments can help remove incorrect edges, but it is constrained by the initial alignment. We also produced new alignments that were not so constrained, by using the probabilities calculated by the model (equation 2) in place of standard substitution matrix scores and affine gap penalties (see Methods). Tables 5 and 6 demonstrate that these alignments also improve structural accuracy compared to the optimal sequence alignment. In contrast, alignments created using the same procedure, except that the regression model was built using only robustness as a variable, are typically less accurate than the optimal sequence alignment. Using frequency as the only variable results in slightly lower performance than the full model. The model-trimmed and model-produced alignments are similar, but not identical. The average shift score between them is 0.96, but ranges from 0.77 to 1.0. The model-produced alignments have higher shift scores (with the Dali alignment as the reference) in 15 of the pairs (average difference 0.03), while the model-trimmed alignments have higher shift scores in 12 of the pairs (average difference 0.02). The model-produced alignments find novel (i.e. not in the optimal sequence alignment) correct structural edges, which the trimmed alignments cannot consider. The full model finds two or more novel correct edges in 18 of the 66 pairs (average count of these 18 is 7.4, maximum 29). The corresponding numbers for the robustness-only and frequency-only alignments are 40, 7.4, 26, and 11, 9.2, 27, respectively. The number of new correct edges found is modest, but there is no disadvantage to using the model-produced alignments compared to the either the optimal alignments or the trimmed alignments, and in some cases using the model-produced alignment provides substantial improvement. These results also support the use of the full model over the frequency-only model. Interestingly, the robustness-only model-produced alignments actually find more novel correct edges than the full model does, even though the overall alignments are clearly poorer. From looking at the alignments and the edges found, it appears that this occurs because the robustness-based alignment produces short runs of 2-6 edges that are structurally correct (typically by placing gaps in different locations), but which do not appear in the optimal or full model-produced alignments (data not shown).

## Discussion and Conclusions

Previous authors have shown that there is substantial overlap between the near-optimal alignment space and the structural alignment space [4,15], and have made use of this information to produce better alignments [15,16] or evaluate alignments [18]. Our results also show this overlap, focusing on statistically significant pairwise alignments (some previous work has tended to focus on more distantly related sequences). It is important to note that the overlap between sequence and structural alignments is due to variation in both spaces: structural alignments produced by different algorithms sometimes vary substantially, in addition to variations in the set of near-optimal sequence alignments. We also emphasize that expectation value is a much more accurate proxy measure for alignment accuracy than the more commonly used percent identity (e.g. [3]). The differences in percent identity between the medium similarity group and low similarity group (defined by E-value) are modest (i.e. both are in the < 30% identity range), but the degree of overlap with structural alignments differs significantly (Figure 3).

Previous efforts to extract information from the overlap between the structural and sequence alignment spaces have used profile-based methods [4,15] to improve the alignments of distantly related proteins; here we focus on pairwise alignments. While profile-based methods are clearly able to detect and align more distantly related proteins than pairwise methods, pairwise alignment is more widely used and in some cases may be the only option available; thus improvements in pairwise alignment accuracy are desirable. Focusing on individual edges in the alignment, Mevissen and Vingron [18] demonstrated that robustness can accurately discriminate between structurally correct and incorrect edges in an alignment; however, they did not produce an explicit model for robustness edge classification, and did not attempt to produce improved alignments. We have developed an explicit model that is more accurate than robustness in predicting whether a given edge (i.e. aligned pair of residues) is likely to be found in a structural alignment, and that produces the associated log-odds probability. Our model can be used to produce alignments that are more similar to structure-based alignments, and is capable of finding correct structural edges that are not in the optimal sequence alignment. The model developed will be integrated into our existing display software that allows users to build and visualize sets of near-optimal alignments, [21,22] making the model easily accessible.

There has also been substantial effort put into developing probabilistic methods for exploring alternative pairwise alignments. We have used the probA program to compare this methodology with the Zuker method of sampling different alignments. It appears that probA samples a more diverse range of alignments, which can result in better agreement with the structural alignments; however, it is not obvious that the "correct" alignments (or edges) are any easier to identify within the set of probA alignments. It is intriguing that in some cases the best probA alignment is also the most similar to the structural alignments (according to the shift score - see Figure 4), which apparently happens rarely if at all with the Zuker alignments. Further investigation will be required to determine if these cases can be distinguished prior to knowledge of the structural alignment.

## Methods

### Near-optimal alignment algorithms

As noted above, the near-optimal solution space can become very large with even small deviations from the optimal score. To accommodate this, several algorithms have been developed that generate samples of the near-optimal space. We chose the Zuker algorithm [8] because it ensures a diverse sampling by forcing all near-optimal edges to be included in at least one alignment while at the same time preserving information about which edges within the set of all edges are used most frequently. The Zuker algorithm identifies all the residue pairs aligned within a sub-optimal range, and produces an alignment that includes those pairs. Because different alignments can have the same suboptimal score, the complete set of alignments, which can be produced by the Waterman-Byers algorithm [6], is very large. The Zuker approach does not guarantee that every possible alignment is produced, but it does guarantee that every possible pair of aligned residues (an alignment edge) is sampled. The maximum number of alignments produced in the 75% of optimal set (within a particular set of scoring parameters) was 44,581, while the minimum was 1. A summary of the numbers of alignments produced for each pair is in Additional File 1.

### probA alignments

We obtained a copy of the probA program [14] from http://www.tbi.univie.ac.at/~ulim/probA/, and used the option to produce 1000 alternative alignments, using either BLOSUM 50 or BLOSUM 62 scoring matrices. probA uses gap open/extension penalties of -9.5/-1.2 for BLOSUM 50, and -7.5/-0.9 for BLOSUM 62. In Figure 2 we used the probA scores; however, when calculating bit scores and robustness values we calculated the sequence similarity scores using the more traditional -10/-2 and -11/-1 penalties.

### Structural alignment programs

The structural alignment programs used were Dali, LSQMAN, CE, and Matras. We used the standalone version of the Dali program [23], called DaliLite [24], obtained from the website ftp://ftp.ebi.ac.uk/pub/contrib/holm/dl/, with default parameters. We used the Linux version of the Combinatorial Extension (CE) program [25], obtainable at http://cl.sdsc.edu/ce.html, also with default parameters. We used the Structal method as implemented in the LSQMAN program [26] from the Uppsala Software Factory: http://xray.bmc.uu.se/usf/. Specifically, we used the Fast Force and Improve commands to get an initial alignment, then the DP command to implement the dynamic-programming method of Levitt and Gerstein [27]. We then used the Global command to calculate the statistics based on the Gerstein and Levitt structural similarity score [27]. For Matras, we used the Linux version of the program provided by the authors [28] with default parameters.

### Alignment comparison metrics

We evaluated sequence and structure alignments using two different metrics: (1) an *individual *metric, such as the Needleman-Wunsch semi-global alignment score (i.e. end gaps are not penalized) [5] or the structural alignment score, and *pairwise *alignment scores, which compare two different alignments. For a structural alignment score we used the Structal score [27] calculated with the LSQMAN program (using the Xalign option) to characterize individual structural alignments. The pairwise metric used was the shift score described by Cline, et al. [15].

### Protein Families

The protein pairs were selected from CATH (version 3.2) domains [29] of known homology and grouped according to expectation values (see Additional File 2). The expectation values were computed using an all vs. all SSEARCH [30] database search using the whole CATH database. We used three groups of sequence pairs, spanning 33 CATH families: high similarity (expectation value (E() < 10^-10^, average percent identity 48.0%), medium similarity (E() < 5 × 10^-5^, average percent identity 26.9%), and low similarity (E() < 2 × 10^-2^, average percent identity 22.6%).

### Logistic Regression

The logistic regression was performed using the R statistical computing system [31]. Models were built using default parameters for the *glm *(generalized linear model) function with the logit link function and the *lrm *(logistic regression model) function from the Design library [32]. The binary response variable for the models was created by using the number of times a particular edge occurred within the set of structural alignments (e.g. if an edge appeared in 2 or more of the 4 structural alignments then the response variable would be set to 1; otherwise it would be set to 0). Three predictors for the model were the robustness of the edge, [18] the frequency of the edge, and the maximum bits-per-position of the edge. Robustness and maximum bits-per-position were normalized to a range of 0 to 1 (in the case of robustness by using the highest and lowest robustness values in a set of suboptimal alignments, rather than within a single alignment).

### Variable Selection

We evaluated the three predictor variables first by building single parameter models using each of the possible predictors. Individual predictors were evaluated following the strategy described in Hosmer and Lemeshow (2000).; any variable with a p-value less than 0.25 was considered for inclusion in the model. (The 0.25 threshold is deliberately large to allow variables that may only be significant when interacting with other variables to be included.) Second, we built a model using all possible predictors and then selectively omitted one or more parameters until the most parsimonious model was achieved. Parsimony was measured with the Akaike Information Criterion (AIC) [33]. Of all the models constructed (using different scoring parameters, edge sample sizes, and target thresholds), all included the frequency, 60% included robustness, and 33% included maximum bits-per-position. Variable omission was due to very small changes in AIC scores; the average difference in the AIC was 0.65 (range 20 to 7264), indicating that the difference between variable selection strategies is minimal. A Kruskal-Wallis test [34] confirmed that the model parameters are robust to changes in sample size, scoring parameters, and target thresholds, indicating that we do not need to construct different models for different combinations of these parameters. Table 2 shows the final model parameters, and Table 3 the analysis of variance for the model. Table 4 shows the residual deviance and AIC values for various combinations of predictor variables.

The final model is represented by the equation:(1)

where *p *is the probability that the edge is contained in a structure-based alignment, *q *is 1-*p*, *r *is the robustness of the edge, *m *is the maximum bits-per-position for an alignment containing that edge, and *f *is the frequency that the edge is seen in an alignment. *a*_1_, *a*_2_, and *a*_3 _are coefficients. Given (1), the probability of an edge being found in a structural alignment is:(2)

### Model assessment

Sequence pairs were partitioned into test and training sets, so that edges from a given sequence pair were not used to both train and test the model. As recommended by Hosmer, et al. [35] we used a smoothed residual test statistic, with the p-value calculated based on a chi-squared distribution (Table 2). Performance was also assessed by plotting ROC curves and comparing the area under the curve (AUC) (Figure 5).

### Model-based alignment generation

We used a modified version of our suboptimal sequence alignment generation code to produce alignments where a diagonal edge in the dynamic programming matrix was scored using the log-odds probability produced by the logistic regression model (Equation 2) in place of the normal substitution matrix score. Gap open/extension penalties of -10/-2 were used. We also constructed alignments using the same method, using only robustness or only frequency to calculate the log-odds probability (Tables 5 and 6).

## Authors' contributions

MLS carried out data collection and analysis, and wrote portions of the manuscript. MES wrote the suboptimal sequence alignment code, carried out data collection and analysis, and wrote portions of the manuscript. EJB participated in the design and coordination of the study and edited the manuscript. WRP conceived of the study, participated in its design and coordination, and edited the manuscript. All authors read and approved the final manuscript.

## Supplementary Material

Additional file 1 **Counts of suboptimal Zuker alignments sampled**. Maximum and minimum number of suboptimal alignments produced for each sequence pair, for the neighborhoods with alignment scores within the indicated percent of the optimal score. Each neighborhood had six different combinations of scoring matrix and gap penalties. Click here for file

Additional file 2 **List of CATH domains used in the study**. The table lists the pair numbering used throughout the paper, the CATH version 3.2 domain names, the expectation value calculated by SSEARCH in a search of a database of 10,000 domains, the percent identity, and the CATH family of the domains. Click here for file
