# Supplementary material for: Improving pairwise sequence alignment accuracy using near-optimal protein sequence alignments
Source: BMC Bioinformatics. 2010 Mar 22;11:146. doi: 10.1186/1471-2105-11-146 (PMC2850363; doi:10.1186/1471-2105-11-146)
Supplement: Additional file 1 — Counts of suboptimal Zuker alignments sampled. Maximum and minimum number of suboptimal alignments produced for each sequence pair, for the neighborhoods with alignment scores within the indicated percent of the optimal score. Each neighborhood had six different combinations of scoring matrix and gap penalties. [file 1471-2105-11-146-S1.PDF]

Additional File 1. Maximum and minimum number of suboptimal alignments produced for each sequence pair, for the neighborhoods with alignment scores within the indicated percent of the optimal score. Each neighborhood had six different combinations of scoring matrix and gap penalties.

	75% of optimal		95% of optimal		100% of optimal	
Pair #	Max	Min	Max	Min	Max	Min
1	23151	13458	4145	1573	160	12
2	15648	9093	2690	1016	64	2
3	15721	9908	2492	1040	2	1
4	12304	6375	1744	404	4	1
5	7485	4906	887	182	3	1
6	21448	9804	4056	1110	32	4
7	7819	4143	1035	206	4	1
8	7768	4058	960	191	12	1
9	4448	2449	370	21	2	1
10	5086	2957	532	37	1	1
11	3903	2039	308	11	1	1
12	6316	3613	758	122	12	3
13	3045	1227	152	28	4	2
14	4202	2316	417	48	1	1
15	2304	1266	114	3	1	1
16	3117	1568	254	78	3	1
17	2737	1331	226	38	6	2
18	3898	2169	402	77	6	2
19	4050	1695	444	38	9	5
20	1999	688	115	35	18	2
21	1830	729	118	17	8	4
22	3507	1274	359	84	60	4
23	8930	837	839	36	11	1
24	3631	742	325	28	6	1
25	3530	759	257	20	4	1
26	8106	1436	1173	39	48	1
27	867	213	55	14	6	1
28	2630	677	335	60	48	8
29	3213	575	197	12	12	1
30	6612	1061	756	33	6	1
31	4166	456	243	21	4	2
32	4959	1554	578	118	32	8
33	21996	2796	3757	140	13441	41
34	5015	1388	560	110	144	12
35	1837	424	173	27	2	1
36	6662	745	691	27	256	2
37	6053	1163	634	65	36	6
38	2642	33	288	12	10	3
39	3242	725	316	16	4	1
40	1532	113	95	16	4	1
41	36976	678	7422	129	3840	8

42	888	232	103	23	16	2
43	214	43	18	8	3	1
44	1496	88	107	9	5	3
45	197	1	6	1	1	1
46	1227	74	114	14	13	2
47	2533	310	280	6	12	1
48	7713	186	437	39	64	4
49	3907	149	439	35	96	6
50	44581	2939	5628	238	65	11
51	637	78	72	11	5	2
52	8510	41	1318	29	2160	8
53	1052	190	54	15	14	2
54	11031	84	833	16	108	2
55	209	2	15	1	3	1
56	2952	76	220	17	16	3
57	169	6	9	2	8	2
58	4986	267	740	25	1918	3
59	16726	687	2318	111	288	6
60	2279	53	189	11	32	1
61	244	28	26	5	12	1
62	3244	77	233	29	20	2
63	1399	5	120	2	8	2
64	3192	12	210	12	12	4
65	3389	19	265	13	24	1
66	1804	6	117	3	10	1
