# Supplementary material for: Improving pairwise sequence alignment accuracy using near-optimal protein sequence alignments
Source: BMC Bioinformatics. 2010 Mar 22;11:146. doi: 10.1186/1471-2105-11-146 (PMC2850363; doi:10.1186/1471-2105-11-146)
Supplement: Additional file 2 — List of CATH domains used in the study. The table lists the pair numbering used throughout the paper, the CATH version 3.2 domain names, the expectation value calculated by SSEARCH in a search of a database of 10,000 domains, the percent identity, and the CATH family of the domains. [file 1471-2105-11-146-S2.PDF]

**Additional File 2** CATH domains used.

| Similarity Group | Pair number | CATH domain pairs | E()-value | Percent Identity | CATH Family |
|---|---|---|---|---|---|
| **High** | 1 | 1cv2A00 1cqwA00 | 0 | 57.0 | 3.40.50.950 |
| | 2 | 1aklA02 1srpA02 | 0 | 51.8 | 3.40.390.10 |
| | 3 | 1qkmA00 1qktA00 | 0 | 48.5 | 1.10.565.10 |
| | 4 | 1cm8A02 1erkA02 | 0 | 42.8 | 1.10.510.10 |
| | 5 | 1b2rA02 1bx0A02 | 1.4e-45 | 52.1 | 3.40.50.80 |
| | 6 | 1eepA00 1b3oA00 | 6.7e-40 | 37.5 | 3.20.20.130 |
| | 7 | 4mdhA02 1bdmB02 | 2.0e-38 | 49.4 | 3.90.110.10 |
| | 8 | 1tmoA04 1eu1A04 | 3.9e-37 | 52.0 | 2.40.40.20 |
| | 9 | 1b56A00 1pmpA00 | 5.9e-37 | 55.6 | 2.40.128.20 |
| | 10 | 1hdaB00 1outB00 | 2.2e-35 | 47.9 | 1.10.490.10 |
| | 11 | 1bylA00 1qtoA00 | 3.7e-35 | 58.5 | 3.10.180.10 |
| | 12 | 1entE02 1aptE02 | 3.7e-33 | 49.4 | 2.40.70.10 |
| | 13 | 2gtnA01 1jnkA01 | 7.4e-30 | 56.3 | 3.30.200.20 |
| | 14 | 1cl7H00 1ae6H01 | 1.9e-28 | 48.9 | 2.60.40.10 |
| | 15 | 1frrA00 1fxiA00 | 5.8e-28 | 58.3 | 3.10.20.30 |
| | 16 | 1nfiC01 1iknC00 | 4.1e-26 | 42.6 | 3.30.200.20 |
| | 17 | 1rdsA00 1fusA00 | 1.0e-24 | 54.6 | 2.60.40.450 |
| | 18 | 1ce7B02 2aaiB02 | 1.1e-24 | 51.6 | 3.10.30.50 |
| | 19 | 1bhxB00 1yphC00 | 9.5e-18 | 34.7 | 2.40.10.10 |
| | 20 | 1svyA00 1d0nA02 | 1.3e-15 | 36.0 | 3.40.20.10 |
| | 21 | 1etpA01 1cnoG00 | 1.4e-14 | 44.1 | 1.10.10.90 |
| | 22 | 1cl7H00 2hmiD01 | 1.0e-10 | 27.3 | 2.40.10.20 |
| **Medium** | 23 | 1qkmA00 1dkfB00 | 1.3e-10 | 19.9 | 2.60.40.10 |
| | 24 | 1jafA00 2ccyA00 | 1.8e-10 | 36.4 | 1.10.565.10 |
| | 25 | 1hdaB00 1mytA00 | 2.1e-10 | 26.8 | 2.40.10.20 |

| | | | | | |
|---|---|---|---|---|---|
| | 26 | 1cm8A02 1agwA02 | 5.8e-10 | 24.6 | 1.20.120.10 |
| | 27 | 1f21D00 1dokA00 | 7.4e-10 | 35.1 | 1.10.490.10 |
| | 28 | 1rdsA00 1rtuA00 | 7.6e-10 | 31.4 | 1.10.510.10 |
| | 29 | 1b56A00 1dc9A00 | 1.1e-09 | 24.4 | 2.40.50.40 |
| | 30 | 1ck4B00 1ao3A00 | 1.6e-09 | 24 | 3.10.30.50 |
| | 31 | 1ihbA00 1awcB00 | 1.6e-09 | 25.9 | 2.40.128.20 |
| | 32 | 1entE02 1psnA02 | 3.7e-09 | 28.2 | 1.25.40.20 |
| | 33 | 1cv2A00 1cqzB03 | 6.6e-09 | 21.6 | 3.40.50.1820 |
| | 34 | 1b2rA02 1amoA04 | 8e-09 | 29.5 | 2.40.70.10 |
| | 35 | 1a47A04 1b90A02 | 8.2e-09 | 34.6 | 3.40.50.950 |
| | 36 | 1auqA00 1ao3A00 | 1.6e-08 | 20.8 | 3.40.50.80 |
| | 37 | 1cd2A00 1vdrA00 | 2.7e-08 | 24.2 | 2.60.40.450 |
| | 38 | 1bhxB00 2pkaA00 | 5.1e-08 | 22.4 | 3.40.50.410 |
| | 39 | 1bbhA00 1cpqA00 | 9.3e-08 | 26.3 | 3.40.430.10 |
| | 40 | 1ao6A05 1uorA03 | 2.2e-07 | 19.5 | 2.160.20.10 |
| | 41 | 2hpdA00 1egyA00 | 2.3e-07 | 19.5 | 1.20.120.10 |
| | 42 | 1bcgA00 1b7dA00 | 9.6e-07 | 33.3 | 1.10.246.10 |
| | 43 | 1cvuA01 1xkbA01 | 6.6e-06 | 38.1 | 1.10.630.10 |
| | 44 | 1cqxA02 1krhA02 | 5.1e-05 | 24.3 | 2.40.30.10 |
| **Low** | 45 | 3sxlA02 1b7fA01 | 0.00017 | 25.6 | 2.10.25.10 |
| | 46 | 2gtnA01 1vr2A01 | 0.0002 | 29.6 | 3.30.200.20 |
| | 47 | 1cl7H00 2rheA00 | 0.00051 | 26.7 | 2.60.40.10 |
| | 48 | 1cm8A02 1ckjA02 | 0.00056 | 19.6 | 1.10.510.10 |
| | 49 | 1b2rA02 1ndhA02 | 0.00068 | 21.3 | 3.40.50.80 |
| | 50 | 1rmgA00 1czfA00 | 0.00081 | 21.1 | 2.160.20.10 |
| | 51 | 1f21D00 1qe6D00 | 0.00092 | 25.7 | 2.40.50.40 |
| | 52 | 1aklA02 1cglA00 | 0.00096 | 22.4 | 3.40.390.10 |
| | 53 | 1etpA01 1fcdC01 | 0.0012 | 26.4 | 1.10.10.90 |

| | | | | | |
|---|---|---|---|---|---|
| | 54 | 1qkmA00 2prgB00 | 0.0021 | 20.2 | 1.10.565.10 |
| | 55 | 3sxlA02 1ha1A02 | 0.0022 | 17.1 | 3.30.70.330 |
| | 56 | 1hdaB00 1hbiA00 | 0.0026 | 22.3 | 1.10.490.10 |
| | 57 | 1d0gR01 1extB01 | 0.0028 | 17.2 | 2.10.50.10 |
| | 58 | 1entE02 1mppA02 | 0.0039 | 22.9 | 2.40.70.10 |
| | 59 | 1cv2A00 1a7uA00 | 0.0040 | 21.8 | 3.40.50.950 |
| | 6 | 1ce7B02 1abrB01 | 0.0041 | 21.8 | 2.80.10.50 |
| | 61 | 1cvuA01 1autL01 | 0.0046 | 29.2 | 2.10.25.10 |
| | 62 | 1mpyA02 1dhyA02 | 0.0061 | 23.7 | 2.40.10.20 |
| | 63 | 1svyA00 1d0nA04 | 0.0082 | 21.3 | 3.10.180.10 |
| | 64 | 1tmoA04 1fdiA04 | 0.0095 | 22.3 | 3.40.20.10 |
| | 65 | 1aqzB00 1rtuA00 | 0.013 | 21.9 | 2.40.40.20 |
| | 66 | 1nfiC01 1a02N02 | 0.013 | 18.7 | 2.60.40.450 |
